# Relative Abundance of Transcripts ( *RATs*): Identifying differential isoform abundance from RNA-seq

**DOI:** 10.12688/f1000research.17916.1

**Published:** 2019-02-24

**Authors:** Kimon Froussios, Kira Mourão, Gordon Simpson, Geoff Barton, Nicholas Schurch

**Affiliations:** 1 Division of Computational Biology, School of Life Sciences, University of Dundee, Dundee, DD1 5EH, UK; 2 Centre for Gene Regulation & Expression, School of Life Sciences, University of Dundee, Dundee, DD1 5EH, UK; 3 Division of Plant Sciences, School of Life Sciences, University of Dundee, Dundee, DD1 5EH, UK; 4 The James Hutton Institute, Invergowrie, Dundee, DD2 4DA, UK

**Keywords:** Transcriptomics, Differential Isoform Usage, Transcriptional regulation, Gene regulation, Feature selection, Algorithms, Visualization

## Abstract

The biological importance of changes in RNA expression is reflected by the wide variety of tools available to characterise these changes from RNA-seq data. Several tools exist for detecting differential transcript isoform usage (DTU) from aligned or assembled RNA-seq data, but few exist for DTU detection from alignment-free RNA-seq quantifications. We present the *RATs, *an R package that identifies DTU transcriptome-wide directly from transcript abundance estimates. *RATs* is unique in applying bootstrapping to estimate the reliability of detected DTU events and shows good performance at all replication levels (median false positive fraction < 0.05). We compare *RATs* to two existing DTU tools, *DRIM-Seq* & *SUPPA2,* using two publicly available simulated RNA-seq datasets and a published human RNA-seq dataset, in which 248 genes have been previously identified as displaying significant DTU. RATs with default threshold values on the simulated Human data has a sensitivity of 0.55, a Matthews correlation coefficient of 0.71 and a false discovery rate (FDR) of 0.04, outperforming both other tools. Applying the same thresholds for *SUPPA2* results in a higher sensitivity (0.61) but poorer FDR performance (0.33). RATs and DRIM-seq use different methods for measuring DTU effect-sizes complicating the comparison of results between these tools, however, for a likelihood-ratio threshold of 30, *DRIM-Seq* has similar FDR performance to *RATs* (0.06), but worse sensitivity (0.47). These differences persist for the simulated drosophila dataset. On the published human RNA-seq dataset the greatest agreement between the tools tested is 53%, observed between *RATs* and *SUPPA2*. The bootstrapping quality filter in *RATs* is responsible for removing the majority of DTU events called by *SUPPA2* that are not reported by *RATs*. All methods, including the previously published qRT-PCR of three of the 248 detected DTU events, were found to be sensitive to annotation differences between Ensembl v60 and v87.

## Introduction

High-throughput gene regulation studies have focused primarily on quantifying gene expression and calculating differential gene expression (DGE) between samples in different groups, conditions, treatments, or time-points. However, in higher eukaryotes, alternative splicing of multi-exon genes and/or alternative transcript start and end sites leads to multiple transcript isoforms originating from each gene. Since transcripts represent the executive form of genetic information, analysis of differential transcript expression (DTE) is preferable to DGE. Unfortunately, isoform-level transcriptome analysis is more complex and expensive since, in order to achieve similar statistical power in a DTE study, higher sequencing depth is required to compensate for the expression of each gene being split among its component isoforms. In addition, isoforms of a gene share high sequence similarity and this complicates the attribution of reads among them. Despite these challenges, several studies have shown that isoforms have distinct functions ^1– 3^ and that shifts in individual isoform expression represent a real level of gene regulation ^4– 7^, suggesting there is little justification for choosing DGE over DTE in the study of complex transcriptomes.

**Figure 1.**
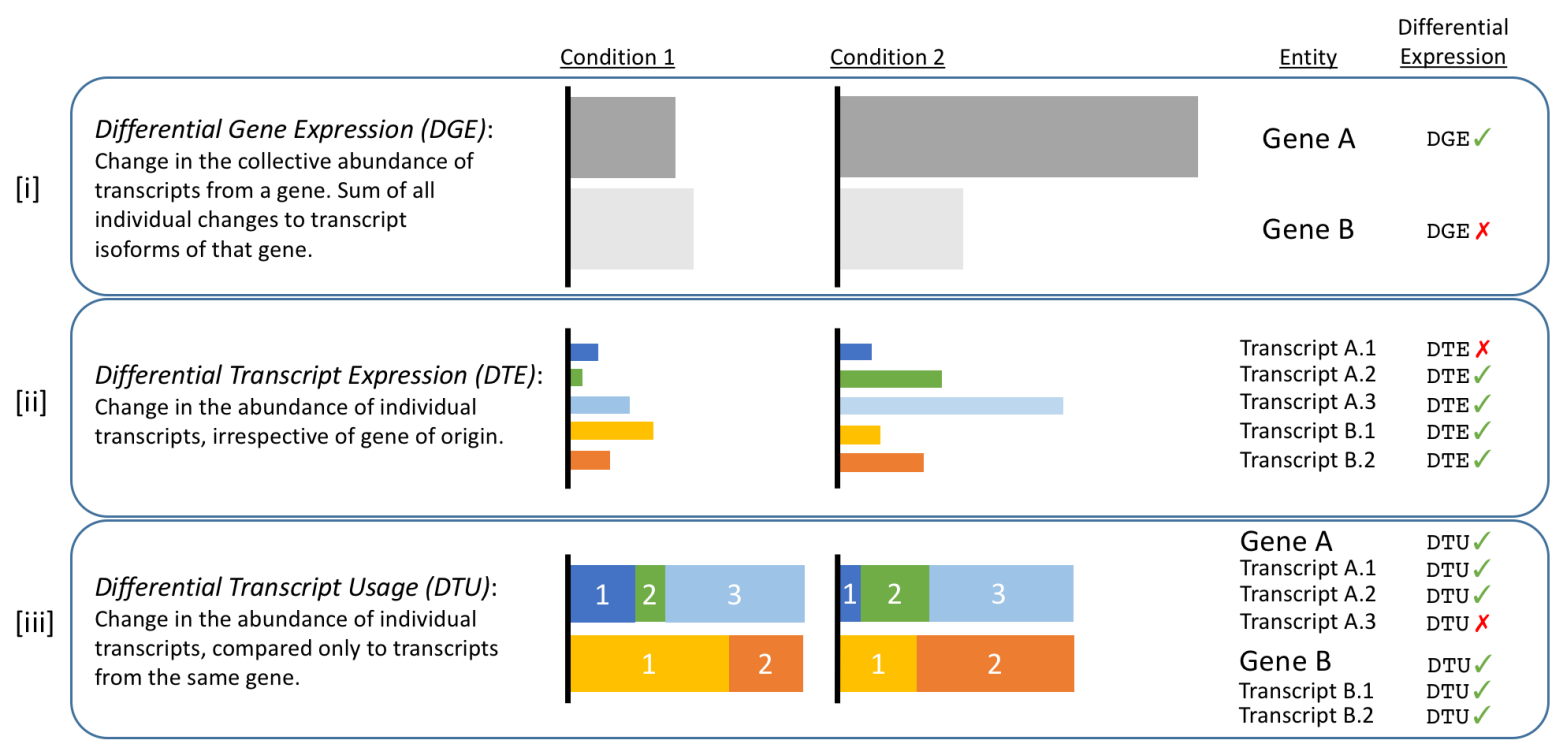
Illustrative definitions of the three types of differential expression analysis (DGE, DTE and DTU). The expression of two genes (Gene A and Gene B), with 3 and 2 isoforms respectively, is compared across two conditions (Condition 1 and Condition 2). The horizontal width of each coloured box represents the abundance of the relevant gene or transcript. A negative differential expression result (red cross-mark) for a given entity in any one of the three analysis types does not exclude that same entity from having a positive result (green tick-mark) in one of the other two analysis types. The relative isoform abundances in [ **iii**] are scaled to the absolute isoform abundances in [ **ii**], which in turn are scaled to the gene expressions in [ **i**]. Gene A is differentially expressed, but only two of its three isoforms are differentially expressed (A.2 and A.3). Proportionally, Gene A’s primary isoform (A.3) remains the same, but the ratios of the two less abundant isoforms change. Gene B is not differentially expressed, but both its isoforms are differentially expressed, and demonstrate an example of isoform switching. DGE: Differential gene expression, DTE: Differential transcript expression, DTU: Differential transcript usage.

It is possible to find significant DTE among the isoforms of a gene, even when the gene shows no significant DGE. This introduces the concept of differential transcript usage (DTU), where the abundances of individual isoforms of a gene can change relative to one another, with the most pronounced examples resulting in a change of the dominant isoform (isoform switching). The definitions of DGE, DTE and DTU are illustrated in Figure 1.

To quantify the isoforms and assess changes in their abundance, most existing tools for DTE and DTU analysis (e.g. *Cufflinks* ^8^, *DEXSeq* ^9^, *LeafCutter* ^10^) rely on reads that either span splice-junctions or align to unique exons. However, with the newest generation of transcript quantification tools ( *Kallisto* ^11, 12^, *Sailfish* ^13^, *Salmon* ^14^), reads are aligned to neither the transcriptome nor the genome. Instead, these tools combine a pseudo-mapping of the k-mers present within each read to the k-mer distributions from the transcriptome annotation with an expectation maximization algorithm, to infer the expression of each transcript model directly. Such alignment-free methods are much faster than the traditional alignment-based methods ( *RSEM* ^15^, *TopHat2* ^16^, *STAR* ^17^) or assembly-based methods ( *Cufflinks* ^8^, *Trinity* ^18^), making it feasible to repeat the process many times on iterative subsets of the read data and, thus, quantify the technical variance in the transcript abundance estimates. However, the lack of alignments prevents these new methods from being compatible with differential expression methods such as *Cufflinks*, *DEXSeq* and *Leafcutter*. Instead, *Sleuth* ^19^ is a tool that handles DTE analysis from alignment-free transcript quantifications. DTU analysis is currently less straight-forward. *SwitchSeq* ^20^ focuses on a particular subset of DTU analysis from alignment-free data, namely isoform switching, whereas *iso-kTSP* ^6^ identifies both DTU and isoform switching, but focuses on the highest-ranking pair of change-exhibiting isoforms per gene. *SUPPA* ^21, 22^, on the other hand, primarily identifies differential splicing events at the junction level, with recent developmental versions having added isoform-level capability. Finally, *DRIM-Seq* ^23^ identifies DTU directly from quantification data, but defines the effect size as a fold change which may not be the most appropriate way to compare proportions.

In this paper, we present *RATs* (Relative Abundance of Transcripts), an R package for identifying DTU directly from isoform quantifications. It is designed to use alignment-free abundance data and is the only tool that exploits bootstrapping to assess the robustness of the DTU calls. *RATs* provides raw, summary and graphical results, allowing for ease of use as well as for advanced custom queries, and the R language is the environment of choice for many widely-used DGE and DTE tools, allowing for easy integration of *RATs* in existing workflows. We assess the accuracy of *RATs* in comparison to *SUPPA2* and *DRIM-Seq* and find *RATs* to perform at as well as or better than its competitors. Finally, we demonstrate that the results of both RNA-seq based and qRT-PCR based analyses are sensitive to the annotation used for transcript quantification and primer design, respectively.

## Methods

### DTU calling

*RATs* identifies DTU independently at both the gene and transcript levels using an efficient implementation of the G-test of independence ^24^, without continuity corrections. The criteria *RATs* uses to identify DTU are described in detail below.

### Pre-filtering

Prior to statistical testing by either method, *RATs* first filters the input isoform abundance data to reduce both the number of low quality calls and the number of tests carried out. Specifically: (i) isoform ratio changes can only be defined for genes that are expressed in both conditions, with at least two isoforms detected, and (ii) transcript abundances must exceed an optional minimum abundance threshold. Transcripts with abundances below the threshold are considered as not detected.

### Statistical significance

Significant changes in relative transcript abundance are detected using two separate approaches: one at the gene level and the other at the transcript level. At the gene level, *RATs* compares the set of each gene’s isoform abundances between the two conditions to identify if the abundance ratios have changed. At the transcript level, *RATs* compares the abundance of each individual transcript against the pooled abundance of its sibling isoforms to identify changes in the proportion of the gene’s expression attributable to that specific transcript. Both methods include the Benjamini-Hochberg false discovery rate correction for multiple testing ^25^. These tests are performed on the summed abundance of each isoform across the replicates.

### Effect size

Transcripts whose absolute difference in isoform proportion is below a set threshold are rejected, even if the difference is statistically significant.

### Reproducibility

*RATs* provides the option to use the bootstrapped abundance estimates obtainable from alignment-free quantification tools to apply a reproducibility constraint on the DTU calls, by randomly selecting individual quantification iterations from each replicate and measuring the fraction of these iterations that result in a positive DTU classification. Typically, each sample is represented by the mean abundance of each transcript, calculated across the quantification iterations. However, this loses the variance information of the quantification. By referring back to the quantification iterations, *RATs* highlights cases where the quantification was unreliable due to high variability and therefore the DTU result should also be considered unreliable. Similarly, *RATs* optionally also measures the reproducibility of the DTU results relative to the inter-replicate variation by iteratively sub-setting the samples pool.

### Implementation

*RATs* is implemented in R ^26^ and has been freely distributed through Github as an R source package since August 2016. *RATs* accepts as input either a set of R tables with abundances (with or without bootstrap information), or a set of *Salmon* ^14^ or *Kallisto* ^11^ output files. An annotation table mapping the correspondence between transcript and gene identifiers is also required, either provided directly or inferred from a GTF file. Results are returned in the form of R *data.table* objects ^27^. Along with the DTU calls per transcript and gene, the tables record the full provenance of the results. Convenience functions are provided for summary tallies of DTU and isoform-switching results, for ID retrieval, and for visualization of the results via ggplot2 (v2.2.1) ^28^. Details on these are available through the user manual of the package. Once created, all plots produced by *RATs* remain customisable via standard ggplot2 operations.

### Performance

The performance was assessed in two ways. Firstly, the false positives (FP) performance of *RATs* (v0.6.2) for detection of DTU between two groups relative to the level of experimental replication was measured on groups generated by random selection without replacement from a pool of 16 high-quality wild-type Colombia-0 *Arabidopsis thaliana* replicates ^29^ ^1^. This was iterated 100 times for each replication level in the range 3 ≤ n ≤ 8. As the two groups are drawn from the same condition, any positive DTU calls must be considered to be false positives. For each iteration, we recorded the fraction of genes and transcripts that were reported as DTU, relative to the total number of genes or transcripts tested in that iteration. The commands and scripts used are from the RATs Github repository.

Secondly, two simulated datasets ^30^ were used to benchmark the sensitivity ( *s*, the fraction of the 1000 DTU events actually detected), false discovery rate (FDR, the fraction of reported DTU events that is not part of the 1000 “real” events) and Matthews correlation coefficient (MCC) of *RATs*, *SUPPA2* and *DRIM-Seq*. The datasets were made of simulated RNA-seq reads based on the transcriptome annotation and to match realistic RNA-seq transcript expression values. To create the second condition, the abundance values of the two most abundant transcript isoforms originating from a gene locus were swapped for 1000 well-expressed coding gene loci. The transcriptome annotation used for both Human and fly comprised only annotated protein coding genes (13937 in the Drosophila, 20410 in the human) leaving a number of other classifications of gene unaccounted for (1745 in the Drosophila, 41483 in the human). These genes constitute a convenient negative set for simulation and should have no expression, save for any reads misallocated to them by the quantification tools. The simulated datasets were obtained from ArrayExpress ^2^ and quantified with both *Kallisto* (v0.44; ^11^ and *Salmon* (v0.9.1; ^14^ using the respective complete annotations that match the simulation of the datasets (Ensembl v70 for the Drosophila and Ensembl v71 for the human; ^30^). The sensitivity, FDR and MCC were measured for a range of comparable parameters between *RATs* (v0.6.4), *SUPPA2 (v2.3)* and *DRIM-Seq ( v1.6, Bioconductor v3.6, R v3.4)*. No transcript abundance pre-filter was imposed on any of the three DTU tools, and the significance level was set to 0.05 for all runs. For *RATs* and *SUPPA2*, three thresholds for the effect size (difference in proportion) were tested; the *RATs*’ current default of 0.2, and more permissive values 0.1 & 0.05. For *DRIM-Seq*, threshold values of the likelihood ratio were explored from 0-30. Finally, *RATs* reproducibility thresholds were explored in the range of 0.8-0.95 for the quantification reproducibility and 0.55-0.85 for the inter-replicate reproducibility. The tool performance was measured using annotations comprised of all annotated genes and only protein coding genes.

### Comparison on a real 2-condition dataset

To test the ability of *RATs* to identify known instances of DTU, we compared it against validated instances of DTU from publicly available RNA-seq data. We took read data from Deng *et al.* (2013, 31), who identified non-DGE changes in the isoform levels of genes between three human patients with idiopathic pulmonary fibrosis (IPF) and three lung cancer patients used as controls. The dataset contains 25 million 54-base long single-end Illumina reads per lung tissue sample. As in the original at study, we used Ensembl v60 ^32^ as the source of the reference human genome and its annotation, in which each of the three discussed genes features two isoforms. Unlike the original study, we used *Salmon* (v0.7.1, with sequence bias correction enabled, 100 bootstrap iterations and default values for the remaining parameters, using k=21 for the index) to quantify the isoform abundances. DTU was identified by *RATs* v0.6.2. For comparison, we repeated the quantification and DTU analysis of the data with the same tool versions and parameters, but using the annotation and assembly from Ensembl v87, the current version at the time of this study.

We also submitted the quantification data to *SUPPA2*, in its *psiPerIsoform* mode, and to *DRIM-Seq*. For a fair comparison, we tried to minimize variability in the parameters and data type used by the three tools. As *SUPPA2* offered no abundance pre-filtering, *RATs* and *DRIM-Seq* were run with abundance threshold values of 0. The p-value cut-off was set at 0.05 for all three tools, using the corrected p-values where available. For the difference in isoform proportion ( *SUPPA2* and *RATs*) the threshold was set at 0.20. No threshold was set for the fold-changes in *DRIM-Seq*. *SUPPA2* required and was provided with TPM abundances. For consistency in the use of abundances normalised for transcript length, *RATs* and *DRIM-Seq* were also provided with TPM, but the values were scaled up to the average library size of 25M reads, as their testing methods expect counts and would be under-powered if used directly with TPMs. Again, the commands and scripts used are available from the RATs Github repository.

## Results

### False positives performance

**Figure 2.**
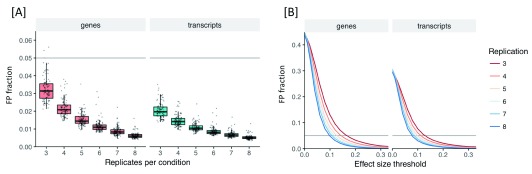
False positives (FP) performance of *RATs* as a function of replication level. False positive fraction measured over 100 permutation iterations of randomly selected (without replacement) replicates from a pool of 16 high-quality wild-type Colombia-0 *Arabidopsis thaliana* replicates from Froussios *et al.* (2017, ^29^). [ **A**] FP fraction of each bootstrap iteration, for default values of all *RATs* parameters (v0.6.2), across a range of replication levels, separately for the gene-level test (red) and transcript level test (blue). [ **B**] Mean FP fraction by replication level, as a function of the effect size threshold (effect size = difference between conditions of an isoform’s proportion). For a gene, the effect size is defined as the largest proportion difference observed among that gene’s isoforms. In every iteration, the FP fraction was calculated against the number of genes or transcripts that were eligible for testing each time (a number which remains very stable across iterations and replication levels – see Extended data 1 ^33^).

Both the gene-level and transcript-level approaches to identifying DTU implemented in *RATs* achieved a median FP fraction <0.05 on our *A. thaliana* dataset, even with only three replicates per condition ( Figure 2A). Higher replication results in both a reduction in the number of false positives and restricts the false positives to smaller effect sizes ( Figure 2B). The gene-level and transcript-level approaches, however, have different strengths and weaknesses. Simultaneously utilizing the expression information across all the isoforms in a gene makes the gene-level test sensitive to smaller changes in relative expression, compared to testing transcripts individually, but it also makes the gene-level test more prone to false positives. Figure 2 shows that the gene-level test has a higher FP fraction than the transcript-level test, irrespective of replication level or effect size, although the two methods converge for highly replicated experiments or large effect sizes. Furthermore, the gene-level test only identifies the presence of a shift in the ratios of the isoforms belonging to the gene, without identifying which specific isoforms are affected. The transcript-level test, in contrast, directly identifies the specific isoforms whose proportions are changing and has fewer false positives than the gene-level test. However, considering each isoform independently requires a larger number of tests to be performed, thus resulting in a greater multiple testing penalty.

### Comparative performance on simulated DTU

**Figure 3.**
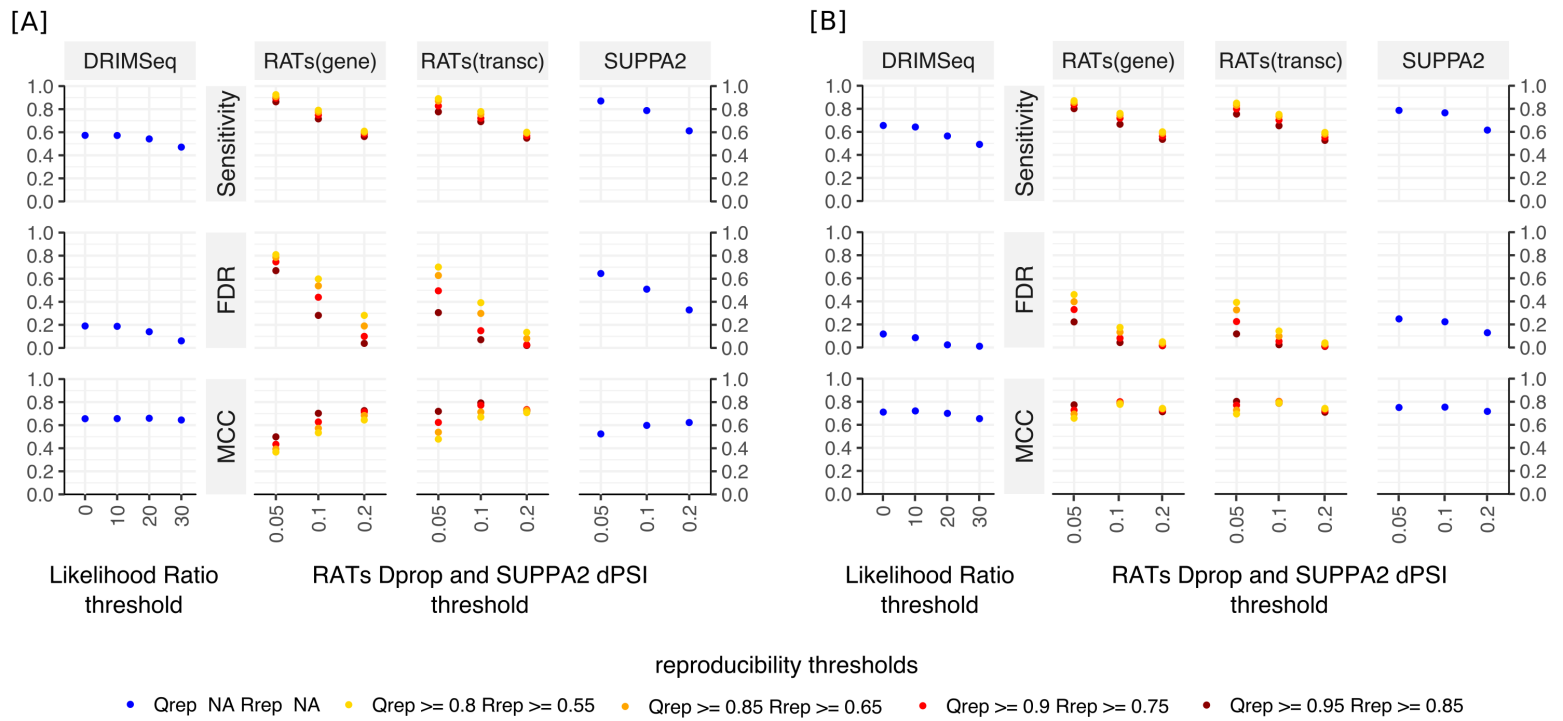
Performance comparison between *RATs*, *SUPPA2* and *DRIM-Seq*. The performance was assessed on the human [ **A**] and Drosophila [ **B**] simulated datasets from ArrayExpress E-MTAB-3766 ^30^, over a range of threshold values for the effect size ( *RATs* - *Dprop*, *SUPPA2* - *dPSI*, *DRIM-Seq* likelihood ratio - *lr*) and confidence in the result ( *RATs* quantification reproducibility – *Qrep*, *RATs* inter-replicate reproducibility - *Rrep*). The statistical significance cut-off was at 0.05 for all cases. The measures of performance are the sensitivity, false discovery rate (FDR) and Matthews correlation coefficient (MCC). The datasets were quantified using *Salmon* 0.9.2 and the metrics were calculated accounting only for the genes strictly listed in the “truth” sets. The results using *Kallisto* for the quantification are practically identical (see Extended data 2 ^33^).

The sensitivity, FDR and MCC performance of *RATs*, *SUPPA2* and *DRIM-Seq* using *Salmon* transcript quantifications of annotated protein coding gene isoforms are summarised in Figure 3. Tested with the simulated Human dataset, the parameter defaults for *RATs* (quantification reproducibility >95%, inter-replicate reproducibility >85% & effect-size >0.2) result in a sensitivity of *s* = 0.55, MCC = 0.71 and FDR = 0.04, outperforming both other tools. With the same thresholds, *SUPPA2* has a higher sensitivity ( *s* = 0.61) but poorer FDR performance (FDR = 0.33). Direct comparison with *DRIM-Seq* is complicated by different methods for measuring DTU effect-sizes between the tools, however for a likelihood-ratio threshold of 30, *DRIM-Seq* has similar FDR performance to *RATs* (FDR = 0.06), but worse sensitivity ( *s* = 0.47). These differences persist for the simulated drosophila dataset. *DRIM-Seq* consistently shows the lowest sensitivity (≤0.65), while maintaining a FDR ≤0.2 in any of the tried parameter sets. *SUPPA2* is the most sensitive of the three tools (0.6 ≤ *s* ≤ 0.9), but also has the highest FDR (0.35 ≤ FDR ≤ 0.65 in human, 0.10 ≤ FDR ≤ 0.25 in Drosophila). *RATs* can match the sensitivity of *SUPPA2* while maintaining a lower FDR than *SUPPA2* by relaxing its quantification reproducibility ( *Qrep)* and inter-replicate reproducibility ( *Rrep)* thresholds. At the highest effect-size thresholds (Dprop _*RATs*_ = 0.2 and *lr _DRIM-Seq_* = 0.3) *DRIM-Seq* has a comparable FDR to that of *RATs*. Surprisingly, the sensitivity, MCC and FDR of *DRIM-Seq* is not strongly sensitive to variations in the likelihood ratio effect-size threshold. Consequentially, RATs has worse FDR performance, but better sensitivity than *DRIM-Seq* at lower effect-size thresholds. Across all the simulated dataset and parameter combinations the gene-level test implemented in *RATs* shows higher sensitivity and higher FDR compared with the results from the transcript-level test. Extending the test to isoforms from the full set of annotated genes, rather than only those from protein coding genes, adds a considerable number of additional true negatives (Drosophila: 1745, human: 4148, see Section: Performance) resulting in a small increase of FDR and slight reduction of MCC for all tools in both datasets (Extended data 2 ^33^). Similarly, using *Kallisto* isoform expression quantifications in place of the quantifications from *Salmon* does not strongly affect the results (Extended data 2 ^33^). The performance results of RATs on these simulated datasets are in good agreement with those presented in Love *et al.* (2018, ^34^), which also demonstrates that the performance of RATs is similar to, or exceeds, the performace of other DTU tools , including DRIM-seq, SUPPA2 or DEX-Seq.

### Recapitulating published validated examples of DTU

After pre-filtering, Deng *et al.* (2013, 31) tested 3098 Ensembl v60 genes for DTU by quantifying their isoform proportions with RAEM ^35^ and using Pearsons Chi-squared test of independence with a FDR threshold of 5%. They identified 248 genes that were not differentially expressed but displayed significant DTU. Subsequently, they confirmed three of them with qRT-PCR: TOM1L1 (ENSG00000141198), CMTM4 (ENSG00000183723), and PEX11B (ENSG00000131779). Table 1 shows the fraction of the 248 DTU genes identified in this study that were also called by *RATs*, *SUPPA2* and *DRIM-Seq*, as well as each tool’s verdict on each of the three validated genes. The genes reported as DTU by *RATs* are listed in Extended data 3 & 4 ^33^ respectively, based on the Ensembl v60 and v87 human annotations.

**Table 1.** comparison of the results by Deng *et al.* (2013, 31) against the results of *RATs*, *SUPPA2* and *DRIM-Seq*, using the same data and annotation (Ensembl v60). The first column shows the fraction of the 248 genes that was recaptured by each method. For methods reporting at the transcript level, results were aggregated to the respective genes. The last three columns show whether the verdicts for each of the validated genes (DTU Yes/No). DTU: Differential transcript usage.

	Deng *et al.* (2013) ^31^	TOM1L1	CMTM4	PEX11B
*RATs* (genes)	0.11	N	Y	N
*RATs* (tr. aggreg.)	0.11	N	Y	N
*SUPPA2* (tr. aggreg.)	0.17	Y	Y	Y
*DRIM-Seq*	0.26	N	Y	N

None of the three tools recapitulated the reported 248 genes well, with the highest fraction of 26% achieved by *DRIM-Seq* possibly due to a tendency to over-predict (see next section). Of the three validated genes, only CMTM4 is reported by all methods, and only *SUPPA2* reports all three genes. Although the rejection of TOM1L1 and PEX11B by *DRIM-Seq* was due to poor statistical significance, *RATs* reported that the changes found were both statistically significant and of sufficient effect size. Instead, *RATs* rejected the genes on the grounds of poor reproducibility (see Section: DTU Calling).

There have been extensive changes in the human transcriptome annotation since Ensembl v60. We hypothesized that these changes could have a significant impact on the set of genes identified in Deng *et al.* (2013, 31). Table 2 shows that in addition to the new genome assembly, the human transcriptome complexity has increased significantly from Ensembl v60 to the more recent v87. Changing the version of the human annotation from Ensembl v60 to v87 removes 10,253 gene IDs and adds 15,839 new ones. Re-quantifying the RNA-seq data with the updated annotation and re-calling DTU resulted in similarly poor overlap between the tools’ results and the original report (see Extended data 5 ^33^). Of the three validated genes, TOM1L1 was unanimously rejected by all methods, CMTM4 remained unanimously reported as DTU, and PEX11B was reported as DTU by *RATs* and *SUPPA2*, but not by *DRIM-Seq*.

**Table 2.** Expansion of the human annotation between Ensembl v60 and v87. In total, the later annotation contains 25% more transcript models. The three genes identified by Deng *et al.* (2013, 31), TOM1L1, CMTM4 and PEX11B, have all acquired additional isoform models.

Human Annotation	Number of transcripts			
	Total	TOM1L1	CMTM4	PEX11B
Ensembl v60 / GRCh37	157,480	2	2	2
Ensembl v87 / GRCh38	198,002	23	5	3

**Figure 4.**
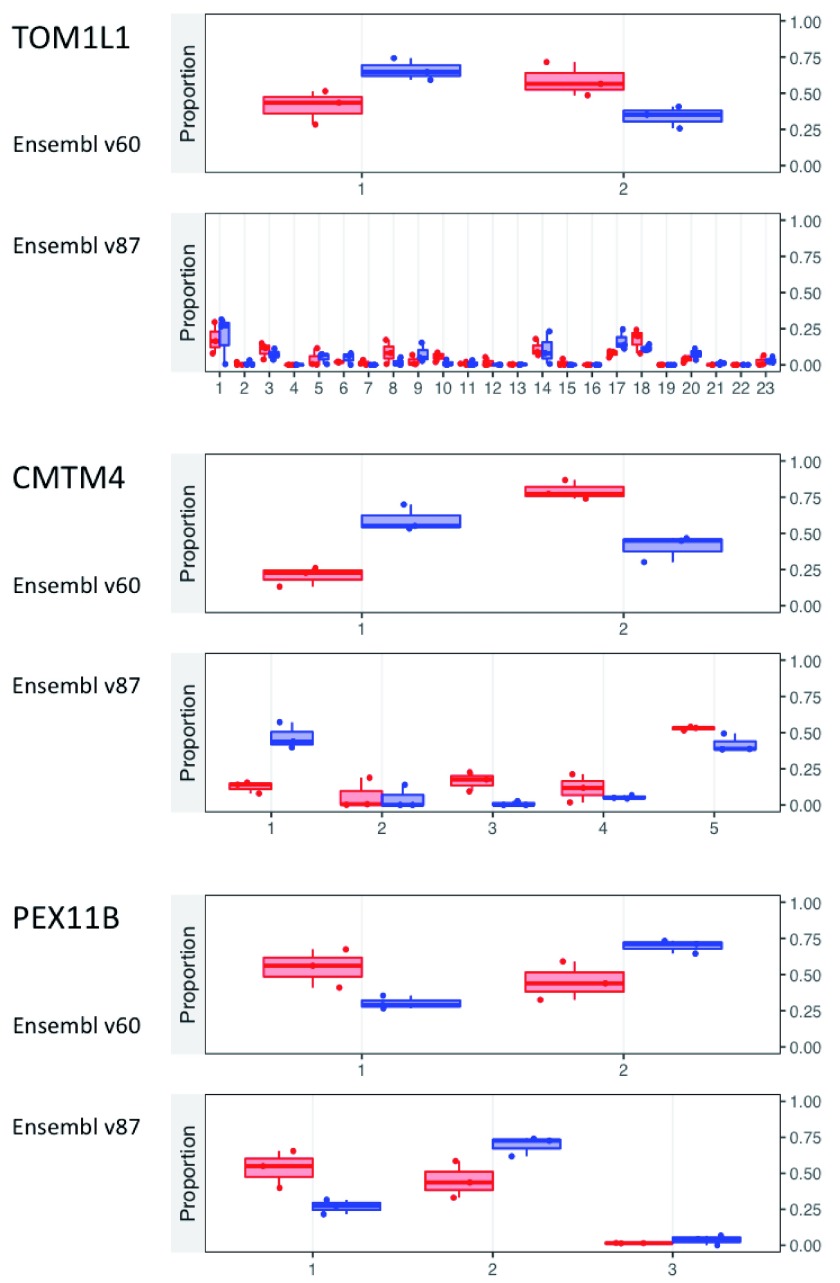
Relative abundance of isoforms for the three validated genes from 31, as re-quantified with *Salmon* 0.7.1 ^14^ using two versions of the Ensembl annotation. Isoform IDs on the *x* axis were replaced with simple numbers to minimize clutter, but the mapping of number to ID is maintained between the two annotations. The *y* axis represents the relative abundance of each isoform. In red are the quantifications from the three replicates of the Control condition, and in blue are those from the IPF condition. The full version of the plots by *RATs*, including the full isoform IDs, is available in Extended data 5 ^33^.

The isoform abundances in Figure 4 reveal that all three genes showed plausible shifts in relative isoform abundance with the Ensembl v60 quantifications, but only PEX11B showed the same shift with Ensembl v87. Instead, TOM1L1 showed no significant changes in any of its 23 isoforms and the primary isoform in the Control samples changed from isoform 2 (ENST00000445275) to isoform 1 (ENST00000348161). CMTM4 shows a similar abundance shift with v87 as it did with v60, but the isoforms implicated changed from isoforms 1 (ENST00000330687) and 2 (ENST00000394106) to isoforms 1 and 5 (ENST00000581487). These changes of context raised questions about the qRT-PCR validation performed in the original analysis of the data ^31^. Indeed, when the reported qRT-PCR primers were aligned to the Ensembl v87 sequence and annotation (see Extended data 6 ^33^), only the primers for PEX11B yielded the same conclusion as with Ensembl v60. For TOM1L1, the primers intended for ENST00000445275 no longer matched that isoform, but matched two other isoforms instead (ENST00000570371 and ENST00000575882). Additionally, the primers intended to quantify the gene as a whole failed to match half of the gene’s new isoforms, and the two sets of captured isoforms did not overlap completely and were thus incomparable in any meaningful way. As a consequence, the qRT-PCR intensities measured in the original study are actually impossible to interpret in the context of the updated annotation and the originally reported conclusion is likely wrong. For CMTM4 the primers reported matched multiple but not all isoforms, casting doubt on the interpretation of the qRT-PCR measurements for this gene as well. Only for PEX11B did the primers target the isoforms in a way that would give interpretable results and indeed lead to the same conclusion as originally reported ^31^.

### Comparison of DTU methods against Deng *et al.* (2013, 31)

Table 3 summarises the results obtained by *RATs*, *SUPPA2* and *DRIM-Seq* for the Deng *et al.* (2013, 31) dataset using Ensembl v60 (same as the original study) and Ensembl v87 (current version at time of the present work). With either annotation, *DRIM-Seq* reported the most DTU genes – almost 1000 with v60 and almost 1700 with v87. The *RATs* gene-level method reported fewer genes by a factor of 1.5 and 2 respectively compared to *DRIM-Seq* with each annotation. *SUPPA2* reported several hundred transcripts more than *RATs*, but at the gene level the numbers were comparable. *RATs* and *DRIM-Seq* reported more genes and transcripts with v87 of the annotation than with v60, whereas *SUPPA2* reported slightly fewer with v87. Despite overall similar volume of results between the two versions of the annotation, it is evident from Table 3 that the overlap of the results between annotations is poor for all methods. For *RATs* and *SUPPA2*, only 30–40% of the genes reported with Ensembl v60 were also reported with v87. For *DRIM-Seq* this overlap was 55% of its Ensembl v60 results.

**Table 3.** Summary of DTU features (genes or transcripts) detected by each method. *DRIM-Seq* reports DTU only at the gene level. *SUPPA2* reports DTU only at the individual transcript level. *RATs* reports at both the transcript and the gene levels, using its respective test implementations. For *SUPPA2* and the transcript-level approach in *RATs*, gene-level results can be inferred from the reported transcripts; these are included in the table, enclosed in parentheses. The last two columns show the reproducibility of the results between annotation versions. DTU: Differential transcript usage.

	*RATs* (genes)	*RATs* (transc)	*RATs* (tr. aggr.)	*SUPPA2*	*SUPPA2* (tr. aggr.)	*DRIM-Seq*
*RATs* (genes)		-	97%	-	46%	19%
*RATs* (transc.)	-		-	35%	-	-
*RATs* (tr. aggr.)	78%	-		-	42%	17%
*SUPPA2*	-	53%	-		-	-
*SUPPA2* (tr. aggr.)	42%	-	49%	-		17%
*DRIM-Seq*	39%	-	43%	-	38%	

The overlap of results between different methods is similar to the overlap of results between annotations, as shown in Table 4. 97% of the genes reported by gene-level method in *RATs* are also identified as DTU by the transcript-level method. Among all the pairwise comparisons of *RATs*, *SUPPA2* and *DRIM-Seq*, however, the highest level of agreement at both transcript and gene level is between *SUPPA2* and *RATs*. *SUPPA2* identifies DTU in 53% of the transcripts that are called as DTU by the transcript-level method in *RATs*, however *RATs* calls DTU for only 35% of the transcripts identified as DTU by *SUPPA2*. *DRIM-Seq* consistently reports a higher number of DTU identifications than either *RATs* or *SUPPA2*, but still only manages at most 43% agreement with the other two tools.

**Table 4.** Overlap between the DTU results from *RATs*, *SUPPA2* and *DRIM-Seq*, for quantification of the Deng *et al.* (2013, 31) dataset based on Ensembl v87. The overlaps are shown as the proportion of the results from the methods on the columns captured by the methods on the rows.

	Ensembl v60		Ensembl v87		Overlap (v60 & v87)	
	genes	transc	genes	transc	genes	transc
*RATs* (genes)	673	-	817	-	272	-
*RATs* (transc.)	(553)	772	(652)	833	(213)	223
*SUPPA2*	(780)	1391	(753)	1252	(257)	374
*DRIM-Seq*	987	-	1680	-	541	-

**Figure 5.**
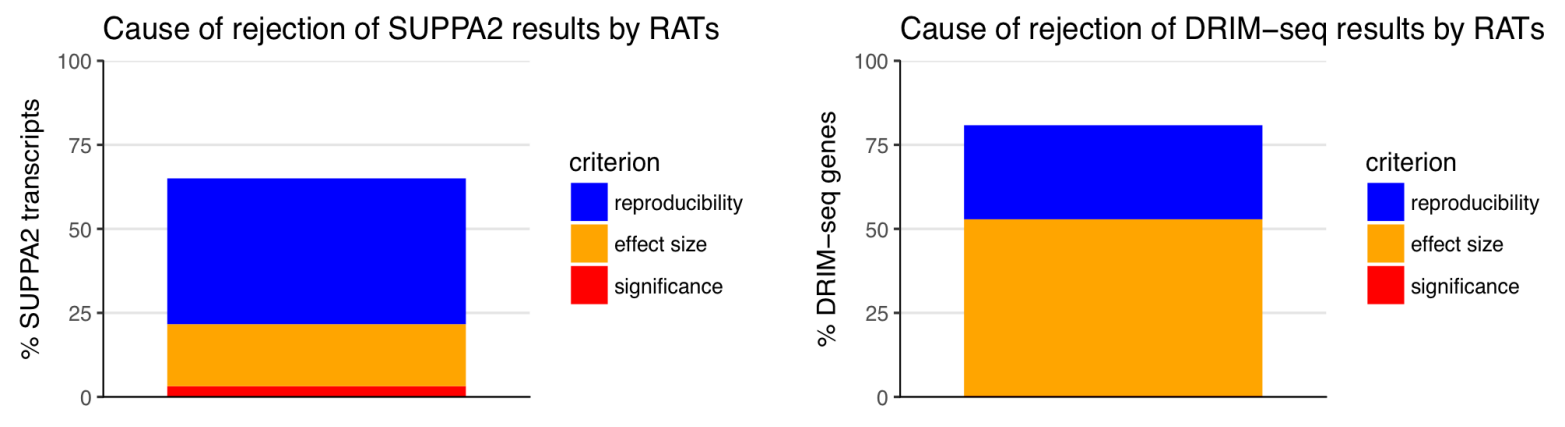
Causes of rejection by *RATs* of results reported by *SUPPA2* or *DRIM-Seq*, expressed as proportion of the total DTU identifications reported by *SUPPA2* (1252 transcripts) or *DRIM-Seq* (1680 genes). The colours represent the different criteria imposed by *RATs*. Since no abundance pre-filtering was enabled for any of the tools, there are no rejections caused by the transcript abundance and the effective number of expressed isoforms. DTU: Differential transcript usage.

*RATs* and *SUPPA2* are more similar than implied by the level of agreement presented in Table 4. Figure 5 shows that the novel reproducibility testing feature in *RATs*, which discounts DTU identification from highly variable quantifications (see Section: DTU Calling), is responsible for rejecting 43% of the *SUPPA2* DTU transcripts and 28% of the *DRIM-Seq* DTU genes that pass the significance and effect size filtering criteria. 53% of the *DRIM-Seq* results and, perplexingly, 18% of the *SUPPA2* results are rejected due to the effect size filter (after passing the significance testing, but prior to the reproducibility filter), despite all the tools operating on the same input isoform quantifications.

### Hardware requirements and run times

*RATs*’ runtime and memory consumption depend on the size of the annotation and the number of bootstraps iterations. Where multiple processing cores are available, *RATs* can be instructed to take advantage of them. The runtime and maximum memory usage for the two simulated datasets from our benchmarks, running on a high-specification laptop, are shown in Table 5.

**Table 5.** Runtime and maximum RAM usage for the Drosophila and human simulated datasets, running on a hyper-threaded quad-core 15” 2015 Macbook Pro with SSD and 16GB RAM. Measured via the *peakRAM* package ^36^. For the bootstrapped runs, 100 iterations were used for the quantification reproducibility and 9 for the cross-replicate reproducibility, representing all the pairwise combinations of the 3 replicates per condition.

Dataset	# of Bootstraps	# of Threads	Wallclock Time (hh:mm:ss)	Max RAM (GB)
Drosophila	0	1	00:00:16	0.36
		8	00:00:08	0.86
	100 + 9	1	00:20:20	1.01
		8	00:07:56	0.87
Human	0	1	00:01:39	3.05
		8	00:00:47	3.21
	100 + 9	1	02:11:13	4.25
		8	00:47:47	4.15

## Discussion

Reliable identification of differential isoform usage depends critically on i) the accuracy of the upstream isoform expression quantifications, and ii) on the accuracy of the annotation they use. *RATs* is the first differential isoform usage tool to include the reproducibility of the upstream isoform expression quantifications to refine its DTU identifications, directly addressing the accuracy of the upstream isoform expression quantifications. Leveraging the bootstrapped isoform expression quantifications from fast modern alignment-free isoform expression quantification tools (such as *Kallisto* and *Salmon*) allows *RATs* to reject those cases of DTU that are based on highly uncertain isoform quantifications. Existing tools rely on the mean isoform abundances, which can hide a large degree of variability, and are thus insensitive to this reproducibility criterion. We recommend running *RATs, and the underlying* alignment-free isoform expression quantification tools that generate the data it operates on, with at least 100 bootstrap iterations.

We evaluated *RATs* on both simulated data and on a high-quality experimental dataset from Deng *et al.* (2013, 31) and show that it outperforms both *DRIM-Seq* and *SUPPA*2. On the simulated data with stringent effect-size, reproducibility and statistical significance threshold, both the gene-level and transcript-level methods in *RATs* have a lower FDR than the other two tools, for a comparable sensitivity and comparable or superior Matthews correlation coefficient. This makes *RATs* particularly useful for data from organisms with large transcriptomes where the risk of false positives is higher. Relaxing these stringent thresholds increases the FDR for all the tools and for the lowest tested effect-size thresholds all the tools struggle to control their FDR adequately leaving little room for optimism regarding the identification of DTU with small effect sizes, particularly in low expression genes. The choice of alignment-free transcript quantification tool did not strongly affect the performance of the DTU tools within the examined parameter space, although in the simulated datasets *Kallisto* appears more prone to overestimating the expression of non-protein-coding genes that in the design of the simulation are not expressed (see Extended data 2 ^33^). Comparing the DTU classifications of the three tools against the instances of DTU identified in the Deng *et al.* (2013, 31) dataset, we found pairwise overlaps between the tools of at most 53%. The low level of agreement between the three tools reflects their different methodological choices, such as the very different definitions of effect size. Both *SUPPA2* and *RATs* use the difference in relative isoform abundance as their measure of the DTU effect size, however *RATs* tests this difference directly whereas *SUPPA2* extrapolates it from the differential inclusion of splice sites. This comparison also highlights the dependence of DTU identification methods on the accuracy of the underlying transcriptome annotation, (a limitation common to all biological tools that use an annotation as guide ^37^). Running *RATs*, *SUPPA2*, and *DRIM-Seq* on the Deng *et al.* (2013, 31) datasets with two different versions of the ensembl *H. sapiens* transcriptome annotation separated by six years produces dramatic differences in the DTU identification results. All three validated DTU genes from the original Deng *et al.* study contained additional isoforms in the newer annotation and only one of these genes displayed the same isoform abundance shifts using both annotations. With the newer annotation, the DTU of one validated gene was attributable to different isoforms depending on the annotation version, while another showed no significant DTU with the newer annotation. qRT-PCR has long considered the *de facto* standard for orthogonal confirmation of high-throughput transcriptomic results however it too is subject to the same limitation, illustrated by multiple matches of the specific primer sequences used for validation in the Deng *et al.* (2013, 31) study in the newer annotation. Annotation of the transcriptomes remains a work in progress even for model organisms and the extensive sequence overlap between isoforms together with the ongoing discovery of additional isoforms suggests that qRT-PCR may not be a suitable method for the validation of transcript abundance changes. For hybridization-based methods like qRT-PCR to serve as a reliable validation method for RNA quantification, the suitability of the primers should first be validated by sequencing the captured amplicons. Soneson *et al.* (2016, ^30^) show that pre-filtering annotations can improve quantification performance and this approach may also be helpful in qRT-PCR primer design.

In the future, experiment-specific transcriptome annotations could be obtained by including a parallel set of full-length isoform RNA-seq data in the experimental design, such as via PacBio sequencing or Oxford Nanopore Direct RNA-seq. An advantage of this approach is that it would better define the transcriptome for the specific experiment ^38– 41^. This may be of importance for experiments focusing on specific tissues or developmental stages of an organism, where the active transcriptome is likely to be only a subset of the global reference transcriptome of the organism.

## Data availability

### Underlying data

The *Arabidopsis thaliana* RNA-sequencing data used in this study is available from ArrayExpress under the study E-MTAB-5446. The simulated *Homo sapiens* and *Drosophila Melanogaster* datasets are available from ArrayExpress under the study E-MTAB-3766. The Deng *et al.* (2013, 31) data are available from the European Nucleotide Archive, or the Short Read Archive, under the study SRA048904.

### Extended data

Extended data are available along with the source code from GitHub and archived with Zenodo

Zenodo: Extended data. bartongroup/RATS: RATs 0.6.5 - R source package, http://doi.org/10.5281/zenodo.2556564 ^33^

Licence: MIT

### Software availability

The RATs R package is open source and available through Github

Source code: https://github.com/bartongroup/ *RATs*.

Archived source code: http://doi.org/10.5281/zenodo.2556564 ^33^

Licence: MIT

## Footnotes

1
https://www.ebi.ac.uk/arrayexpress/experiments/E-MTAB-5446/

2
https://www.ebi.ac.uk/arrayexpress/experiments/E-MTAB-3766/
