# Supplementary material for: Relative Abundance of Transcripts ( RATs): Identifying differential isoform abundance from RNA-seq
Source: F1000Res. 2019 Feb 24;8:213. [Version 1] doi: 10.12688/f1000research.17916.1 (PMC6426083; doi:10.12688/f1000research.17916.1)
Supplement: Peer review history

# Peer review: Reviewer response for version 1

**DOI:** 10.5256/f1000research.19594.r44975

**Published:** 2019-03-18

**Authors:** Michael I. Love

**Affiliations:** 1 Department of Biostatistics, University of North Carolina-Chapel Hill, Chapel Hill, NC, USA

The authors present a new method for detection of DTU from RNA-seq data, which uniquely leverages quantification uncertainty in the form of inferential replicates. I am not aware of other methods specifically designed to detect DTU as opposed to change in total expression level of the gene, which take into account quantification uncertainty. It is therefore a useful contribution to the methods literature. The authors have taken some length to assess their method against other popular methods on real and simulated datasets, and investigating individual genes with qRT-PCR validation in detail.

 I have some concerns about the conclusions from the evidence provided in the article, and additionally have requests for further details about the methods, which should be presented in the article itself.

**Major comments:**

**1)** The methods are not sufficiently described, I have the following questions:

- What is the input to RATs? Is it TPM or counts or scaledTPM? Should the library size differences be removed prior to providing to RATs or does RATs take care of library size differences internally?
- Can the methods described all analyze the same type of experiment, are they all restricted to two-group analyses? Can any of them control for batch effects?
- What are the default pre-filtering and post-filtering settings? What is the default minimum abundance threshold or proportion threshold for an isoform to be considered expressed? What is the default effect size cutoff, and how is it implemented per isoform, per gene? What is the default fraction for determining that evidence of DTU is not substantiated across inferential replicates? Likewise, what default fraction for biological replicate variation?

**2) **I didn't understand why abundance thresholds were not used, as described here, " *No transcript abundance pre-filter was imposed on any of the three DTU tools*," and also " *As SUPPA2 offered no abundance pre-filtering, RATs and DRIM-Seq were run with abundance threshold values of 0.*" 

 As shown in Soneson *et al.* (2016 ^1^) and Love *et al.* (2018 ^2^), performance of a number of DTU methods is greatly improved by filtering out lowly expressed transcripts. It can be inferred from the title of the former paper: " *Isoform prefiltering improves performance of count-based methods for analysis of differential transcript usage*". 

 SUPPA2 does have an abundance pre-filtering option, which was used in Love *et al.* (2018 ^2^):

 " *We enabled a filter to remove transcripts with less than 1 TPM. TPM filtering is a command-line option available during the diffSplice step of SUPPA2 and this greatly improved the running time without loss of sensitivity*".

 From the SUPPA2 manual: " *-th | --tpm-threshold: Minimum expression (calculated as average TPM value within-replicates and between-conditions) to be included in the analysis. (Default: 0).*"

 Given that all the methods have abundance and/or proportion filters available, that filters are recommended by at least two of the three methods in their documentation (DRIMSeq and RATs), and at least two independent review papers (not introducing methods) have shown that abundance and/or proportion filtering improves performance of methods, I can't see why the choice was made to not use filters.

**3)** It is mentioned in the false positive analysis that the median FP fraction was less than 0.05 and a horizontal line is drawn on Fig 2A and B. This is misleading, as the adjusted p-values are being thresholded at 0.05 (I assume), and in a null comparison the rate of false positives from an adjusted p-values should be 0, not 0.05. Drawing or mentioning a 0.05 cutoff would be relevant for the p-values (uncorrected), but has no bearing on the adjusted p-values. This may confuse readers.

**4)** The authors repeatedly refer to the reported effect size in DRIMSeq being an issue for comparison across methods, e.g. " *Direct comparison with DRIM-Seq is complicated by different methods for measuring DTU effect-sizes between the tools*", but this is only an issue to the extent that the authors wish to perform post-hoc filtering on effect size. It is not an issue for null hypothesis testing without post-hoc filtering, because all methods are testing against the null that the underlying proportion of expression across isoforms has the same distribution for control samples and treated samples. However, I agree that for post-hoc filtering, one may want to filter the methods in a similar manner. It should be easy to filter the DRIMSeq results directly on absolute difference in isoform proportion, for example in Love *et al.* (2018 ^2^) we performed post-hoc filtering for DRIMSeq on the SD of proportions across all samples using a 6-line R function.

 As the likelihood ratio statistic should be 1-1 and monotonic with the p-value for DRIMSeq (if the degrees of freedom is constant across genes or transcripts), then I would not compare effect size filtering with likelihood ratio filtering, as the latter is simply filtering the p-value at a lower threshold.

**Minor comments:**

- In the Introduction, the authors state " *there is little justification for choosing DGE over DTE in the study of complex transcriptomes*". The authors imply that gene-level and transcript-level analysis are mutually exclusive analyses, when they are not, and so I would suggest to reword or reconsider this statement. I and others have encouraged assessment of total changes in gene expression (DGE) as well as changes in isoform proportion (DTU), as both may be present in an experiment and both may be of biological importance to the system being studied. DGE has the property that the majority of inferential uncertainty which exists in an RNA-seq sample is removed (because it occurs across isoforms within genes), leaving inferential uncertainty from reads mapping across gene loci, but this property of reduced uncertainty does not preclude a transcript-level analysis. While DTE has advantages, the above sentence claiming that DGE has none overstates a more complex situation in my opinion.
- Throughout the paper, the authors refer to "DRIMSeq" as "DRIM-Seq" which is minor but different than the software and publication.
- For what it's worth, the transcript-level test is similar conceptually to the current implementation of testForDEU() in DEXSeq which compares the expression of each feature to the sum of expression from all other features of the gene (this is also different from the test described in the original DEXSeq publication). Running DEXSeq on transcript estimated counts with testForDEU() was tested on simulated data in Soneson *et al.* (2016 ^1^) and Love *et al.* (2018 ^2^), and so such an approach has some evidence of working well for detection of isoform changes within a gene.
- I didn't understand what was meant by the following: " *the tables record the full provenance of the results*".
- It is stated that, " *The performance results of RATs on these simulated datasets are in good agreement with those presented in Love et al (2018)*". However, this seems to be not clearly the case, which may be due to differences in the simulated data in the two articles, or some other reason. In the present article, DRIMSeq is reported as having lower sensitivity with lower achieved FDR than other methods, SUPPA2 has higher sensitivity and higher FDR, and RATs with various filter thresholds falls in between. In Love *et al.* (2018 ^2^), DRIMSeq had the opposite performance: higher sensitivity but higher FDR relative to SUPPA2 and RATs run with default filters. However interpretation is made difficult by all the filtering options in Figure 3. It would be easier to compare perhaps if an additional supplementary plot to Figure 3 was made with only the default filter thresholds instead of the filter threshold ranges for all methods. The main commonality across the two benchmarks seems to be that RATs can achieve higher sensitivity than SUPPA2 while maintaining the same precision, for the 5% nominal FDR threshold.
- This sentence needs to be made more specific, or else it could be misleading: " *As a consequence, the qRT-PCR intensities measured in the original study are actually impossible to interpret in the context of the updated annotation and the originally reported conclusion is likely wrong.*" Specifically which conclusion is likely wrong? From the analysis, it seemed like there is not a problem with the original qRT-PCR intensities and interpretation for at least one of the three genes.
- Why is it perplexing that " *18% of the SUPPA2 results are rejected due to the effect size filter*". I didn't follow the authors in that statement.
- It is stated, " *Existing tools rely on the mean isoform abundances*...". This implies that the mean of inferential replicates is used for statistical testing. It's perhaps subtly different, other methods are typically using the maximum likelihood estimate, which may be different than the mean of the bootstrap distribution, and different than the mean of the Gibbs sampling distribution. I would just say that other tools do not make use of inferential replication.

I have read this submission. I believe that I have an appropriate level of expertise to confirm that it is of an acceptable scientific standard, however I have significant reservations, as outlined above.

## References

[1] SonesonCMatthesKLNowickaMLawCWRobinsonMD: Isoform prefiltering improves performance of count-based methods for analysis of differential transcript usage. *Genome Biol*.2016;17: 10.1186/s13059-015-0862-3 12 10.1186/s13059-015-0862-3 26813113PMC4729156

[2] LoveMISonesonCPatroR: Swimming downstream: statistical analysis of differential transcript usage following Salmon quantification. *F1000Res*.2018;7: 10.12688/f1000research.15398.3 952 10.12688/f1000research.15398.3 30356428PMC6178912

# Peer review: Reviewer response for version 1

**DOI:** 10.5256/f1000research.19594.r44977

**Published:** 2019-03-04

**Authors:** James P. B. Lloyd

**Affiliations:** 1 ARC Centre of Excellence in Plant Energy Biology, University of Western Australia, Perth, WA, Australia

‘RATs’ is addressing an important problem: how to quantify changes in transcript isoform usage. Other tools, like ‘Sleuth’, address a related problem, which is differential transcript expression. Both RATs and Sleuth take advantage of the bootstrapping data that tools like ‘kallisto’ and ‘Salmon’ generate when quantifying transcript isoform abundance. By taking advantage of bootstrapping, such tools can estimate the technical variation within the data, to better look for differential changes. Transcript isoform usage is often linked to changes in alternative splicing or isoform specific decay rates (e.g. from NMD). Therefore, having a tool to accurately find changes in isoform usage is vital to our ability to address a range of biological problems. 

 I have tried a version of RATs. I found that it was easy to install and easy to use. Being able to install bioinformatics software is no guarantee (Mangul *et al.*, 2018 ^1^). A bonus of RATs is that several figures can be generated from the data within the tool. This was simple to do, but allowed for you to visualize your data in a straightforward but powerful way. This is one of the rare tools that just works and was relatively intuitive and well-documented. 

 The paper uses sensible approaches to compare RATs to other tools, including ‘SUPPA’ and ‘DRIMSeq’ and the authors found that RATs performed at a similar or better level than the other tools. 

 One minor point that could be better explained is how RATs uses the bootstrapping data. Does it use it to simply throw out highly variable genes (decreasing FP rate) or does it help get closer to the true rate of biological variation, thus increasing the true positive rate? 

 In the methods, it would be good to see more explanation on how RATs does its pre-filtering. For example, if a transcript has zero expression in one treatment but a modest to high expression in the other treatment, would RATs keep this transcript or discard it? This would be of interest to people working on RNA decay pathways, such as NMD. 

 One thing that I would love to see is a comparison of RATs to the DEXSeq/DRIMSeq approach used to address differentiation transcript isoform usage (Love *et al.*, 2018 ^2^). This tool appeared to also perform well in the publication of this approach, where they used sim data. Therefore I think a comparison to RATs here, using sim and real data (human) would be appropriate.

I have read this submission. I believe that I have an appropriate level of expertise to confirm that it is of an acceptable scientific standard.

## References

[1] MangulSMosqueiroTDuongDMitchellKSarwalVHillBBritoJLittmanRStatzBLamADayamaGGrieneisenLMartinLFlintJEskinEBlekhmanR: A comprehensive analysis of the usability and archival stability of omics computational tools and resources. *bioRxiv*.2018; 10.1101/452532 10.1101/452532

[2] LoveMISonesonCPatroR: Swimming downstream: statistical analysis of differential transcript usage following Salmon quantification. *F1000Res*.2018;7: 10.12688/f1000research.15398.3 952 10.12688/f1000research.15398.3 30356428PMC6178912

# Peer review: Reviewer response for version 1

**DOI:** 10.5256/f1000research.19594.r44979

**Published:** 2019-02-28

**Authors:** Sophie Shaw

**Affiliations:** 1 Centre for Genome Enabled Biology and Medicine, University of Aberdeen, Aberdeen, UK

Froussios *et al. *have presented here a new tool, RATS, for the identification of differential transcript usage from transcript abundance estimates. RATs was benchmarked and compared to the existing tools DRIM-Seq and SUPPA2 across four different datasets. False positive rate, false negative rate, sensitivity and Matthews correlation coefficient were all measured. When considered as a whole, RATs was found to outperform the other tools. Differing results due to the version of the reference genome used are also discussed. This is a nicely presented manuscript, with well thought out comparisons. The tool will make a good addition to existing RNA sequencing analysis pipelines, especially as the field moves towards alignment free methods. 

 The rationale for the development of this tool is clearly stated, as there are only a few tools which carry out DTU detection from alignment-free RNA-seq quantifications. The majority of existing tools for DTE and DTU are designed for use with alignment- and assembly-based methods. Of the existing tools described, each has specific uses, and RATs has been presented as a broad "differential transcript usage" identification tool. 

 The methods of the analysis have been described well, and overall are technically sound. I would like to see an expansion on the description of the statistical method underpinning RATs. Although G-test of independence is cited, a brief description of what this entails and how it differs from existing tools would aid in the understanding of how the tool functions. 

 However, I have some suggestions concerning the comparison of tools and the datasets selected. With regards to the selection of tools for comparison, SwitchSeq and iso-KTSP are mentioned within the introduction as being able to use transcript abundance estimates, however are not compared to. I assume that this is because they are too specialist in their identification of differential transcript usage and/or isoform switching, but I think the decision to not compare to these tools should be more explicit. The authors have not mentioned the recent pre-print from Cmero et al. (2019) ^1^^ ^which discusses the development of methods for DTU detection from alignment-free datasets using equivalence classes. The paper uses the same simulated datasets for benchmarking of the tool, and should be considered as another tool to compare to RATs. If this is not deemed as an equivalent method, it should at least be discussed in this manuscript. 

 With regards to the datasets tested, the published human data set which is shown here is not directly confirming the accuracy of RATs, as the authors show that the qPCR validation within the original study may be inaccurate, and underlying issues are present due to the reference genome version. Although the dataset is being used to compare RATs to SUPPA and DRIM-Seq, it is not validating the tool. I think that this manuscript would benefit from comparison of the three tools using another "real-life" dataset, which has been validated in some way, to support that RATs is detecting known DTU. 

 Methods for tool development and testing are clearly described, apart from with false positive testing with *A. thaliana* dataset. The authors should include details on how the transcript abundances were produced for this (using Kallisto or Salmon? Any other pre-processing?). All datasets used are publicly available with accession numbers given. Additional data is provided within published links; however, these would benefit from a simple readme file, which explains the contents of each extended data file so the reader doesn't need to search through them. 

 Within the results, it would be nice to see more discussion on the impact of the bootstrapping information used by RATs. I think that this is a really beneficial part of this tool and this has not been demonstrated enough. It should also be made clearer if this bootstrapping information is obtained solely from Salmon/Kallisto or if RATs implements it's own bootstrapping. 

 Although the testing of the simulated datasets does show that RATs outperforms DRIM-Seq and SUPPA2, I don't feel that as it stands you can conclude that the analysis of the published dataset shows this. When comparing to the published findings, SUPPA2 shows better results with confirmation of the qPCR results. As I've mentioned above, I would find another "real-life" dataset for comparison, or simply re-word the conclusion so that this isn't overstated.

I have read this submission. I believe that I have an appropriate level of expertise to confirm that it is of an acceptable scientific standard, however I have significant reservations, as outlined above.

## References

[1] CmeroMDavidsonNOshlackA: Fast and accurate differential transcript usage by testing equivalence class counts: Supplementary figures and tables. *bioRxiv*.2018; 10.1101/501106 10.1101/501106
